# Affective Mobile Language Tutoring System for Supporting Language Learning

**DOI:** 10.3389/fpsyg.2022.833327

**Published:** 2022-03-24

**Authors:** Chih Hung Wu, Hao-Chiang Koong Lin, Tao-Hua Wang, Tzu-Hsuan Huang, Yueh-Min Huang

**Affiliations:** ^1^Department of Digital Content and Technology, National Taichung University of Education, Taichung, Taiwan; ^2^Department of Information and Learning Technology, National University of Tainan, Tainan, Taiwan; ^3^Department of Engineering Science, National Cheng Kung University, Tainan, Taiwan

**Keywords:** mobile affective tutoring system, asynchronous discussion forum, emotion, collaborative learning, learning performance, usability

## Abstract

Students often face difficulties and experience negative emotions toward second language learning. The affective tutoring system (ATS) is a next-generation learning approach that can detect the affective status of learning to increase performance. Therefore, for the purposes of this study, an innovative affective mobile language tutoring system (AMLTS) was designed to support Japanese language learning. The effects of AMLTS, along with asynchronous discussion, that were intended to improve performance, were examined using a triangulation method. To investigate the effect on emotion, the proposed AMLTS provides a virtual emotion agent that can interact with users and record emotional events, learning assessments, and the results of the interaction into a database. Learning effectiveness evaluations were conducted *via* two experiments: prototype evaluation and final evaluation. Sixty-three students, all beginners, were invited to use the AMLTS to learn Japanese. The research results show that the proposed AMLTS affective interaction design significantly improves learner engagement and performance. In the emotion feedback analysis and learning process, AMLTS helped students deepen their understanding of the content, enabled them to clearly understand the content, and to engage in peer interaction and experience positive emotions. In the evaluation of system usability, AMLTS reveals good usability for foreign language acquisition.

## Introduction

Emotions play a significant role in everyday activities, in turn influencing attitudes, memory, decision making, attention, learning, and learning achievement (Sionti et al., 2018). By assessing the emotions of students, teachers can effectively change their approach to teaching and evaluation, while supporting learning performance (Moridis and Economides, 2008; Hwang and Yang, 2009; Petrovica et al., 2017; Daouas and Lejmi, 2018; Wronowski et al., 2019).

Anxiety around learning a foreign language is a common negative emotion that is experienced while attempting to learn a second language, and it influences both learning outcomes and motivation (Wu et al., 2014). In addition to conducting research on language learning, one study also investigated emotions by employing different methods and information technologies to facilitate teaching and transform an affective tutoring system (ATS) from the system interface of a single computer into online learning courses. The study also employed affective computing to investigate user satisfaction, learning outcomes, variations in the learning experience, and changes in emotional states experienced during in the learning process (Lin et al., 2014).

Interactive discussion provides learners with opportunities to express their thoughts and feelings freely and grants them autonomy while learning, in turn improving motivation, learning outcomes (Chen et al., 2018; Delahunty, 2018), and language use (Lyons et al., 2018).

Therefore, the gaps in research exist in understanding the effects of affective factors associated with language learning, learning performance, and interactive/collaborative learning in ATS to support second language learning. The purpose of this study is to prove the effectivity of a new affective mobile language tutoring system (AMLTS) combined with the ATS, which was designed to be used with a mobile device to investigate learning performance.

## Literature Review

### The Affective Tutoring System

The ATS is a next-generation learning approach that has approved intelligent tutoring systems (ITSs) to detect the affective status of learning and adapt accordingly to increase learning performance (Hasan et al., 2020). Related studies have reported that the effective use of ATS can increase learning motivation and improve learning outcomes (Moridis and Economides, 2008; Sarrafzadeh et al., 2008; Lin et al., 2014, 2020; Moga et al., 2014; Daouas and Lejmi, 2018; Sionti et al., 2018; Wang and Lin, 2018; Hasan et al., 2020). Previous studies have examined the influence of ATS on learning performance during remedial accounting instruction (Sionti et al., 2018) and mathematics (Mastorodimos and Chatzichristofis, 2019). ATS can attract attention, motivate learners, and significantly improve learning performance (Lin et al., 2014; Mehdi et al., 2015; Wang and Lin, 2018; Bakeer and Abu-Naser, 2019).

### Mobile Learning and Asynchronous Discussion Forum

Online asynchronous discussion forums (ADFs) enable effective communication and the exchange of ideas and information, thus improving learning outcomes (Hammond, 2000; Yu, 2001; Oztok et al., 2013). ADFs, which are learner-centered cooperative activities, can facilitate peer learning, increase learning benefits and facilitate interaction, increase user interest, facilitate the sharing of experiences, and maximize learning outcomes (Hammond, 2000; Yu, 2001). ADFs can also help learners increase their learning outcomes and participation (Cheng et al., 2011; Chang et al., 2013; Zheng and Warschauer, 2015).

In summary, mobile learning can help students improve both their learning outcomes and motivation. Different courses and pedagogical strategies can be designed for learners and applied to an ATS, which then provides real-time information to teachers regarding the emotional status of students as a reference point for tutorials. The system developed for the purpose of this study was adopted (1) to investigate whether it can positively influence learning outcomes and usage levels and (2) to obtain learner feedback related to using AMLTS.

## Materials and Methods

### System Design

**Figure 1:**
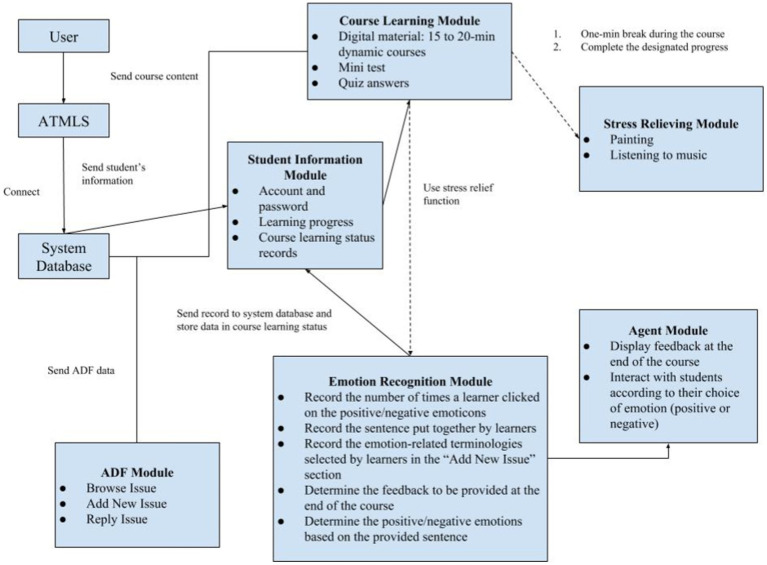
Design of the AMLTS.

The AMLTS application (app) integrates affective computing and ADF functions designed for language learning. A series of adventures about conquering an island was used as the narrative background to attract language learners in AMLTS. The design of AMLTS is shown in Figure 1.

In the course learning module, a course database was designed to store both static and dynamic digital teaching materials, including audio/video files and PowerPoint slides. The course consisted of beginner and elementary courses in Japanese, titled “A Journey to Learning Japanese.” The goal was to establish a foundation in Japanese, in the aspects of listening, speaking, reading, writing, and cultural understanding. Course ideas were derived from the following books: *Minna no Nihongo* (Elementary I), *Japanese Go Go 1*, *Elementary Japanese 1*, *General Knowledge on Japanese*, and *Japanese for Beginners*. These materials were used to design a range of digital course content, including the origin of Japanese words, rules of writing the *goujon*, rules of Japanese pronunciation, and information about Japanese culture. The language-centered content focused on general knowledge of Japanese, including basic greetings in Japanese, everyday Japanese (e.g., numbers, food, clothing, accommodation, travel, education, and entertainment), and Japanese emotional expressions. After a learner completed a quiz, the system checked the answers immediately and informed the learner of his or her performance on the quiz.

The stress relief module provided two kinds of relaxing activities, painting and listening to music, as students moved from dynamic video courses to static courses during breaks. Music performances and songs (in Japanese) were played to supplement language learning in ways other than those taught within the traditional framework, specifically through the appreciation and analysis of songs.

The emotion recognition module processed emotion-related events that recorded the number of times a learner clicked on positive/negative emoticons during the course and evaluated the sentence composed by the student. Feedback was provided at the end of the course according to the rules of the emotion recognition design as well as the emotions (positive or negative) reflected in the student-composed sentence. The emotion-related terminologies selected by learners in the “Add New Issue” section could be used to express the emotion requested in the ADF module. Thus, when leaving comments, learners had the opportunity not only to express opinions and to ask questions, but also an outlet for their emotions surrounding the experience.

The ADF module enables learners to propose questions regarding the course by selecting new issues or imparting and sharing knowledge about an interesting issue with other learners in the menu of the discussion forum. The learning status of the student can also be examined based on the emotions express in the “Add New Issue” section.

The agent module primarily consists of three characters, a monkey, a sailor, and a shipmaster, which act as learning partners. In this study, the agent serves its function by interacting statically with learners when they click on a positive or negative emotion and provides feedback and suggestions at the end of the course through a dialog window. The characters were configured statically so as not to engage in conversations with learners and therefore encourage concentration during class.

### System Process

Learners can opt for the learning activities of their preference in AMLTS. The course consists of five learning activities, outlined as follows: (1) Task 1: Watch a 15- to 20-min-long video on Japanese. (2) Quiz 1: Complete a quiz administered after Task 1 to evaluate the learning outcomes from Task 1. (3) One-minute-long break: Choose either to paint (by selecting the painting interface on the system) or to listen to music (by selecting music from among the in-built options in the program). (4) Task 2: Browse the content of a static course in Japanese. This task is a continuation of Task 1. (5) Quiz 2: Take a quiz administered after Task 2 to evaluate the learning outcomes from Task 2.

**Figure 2:**
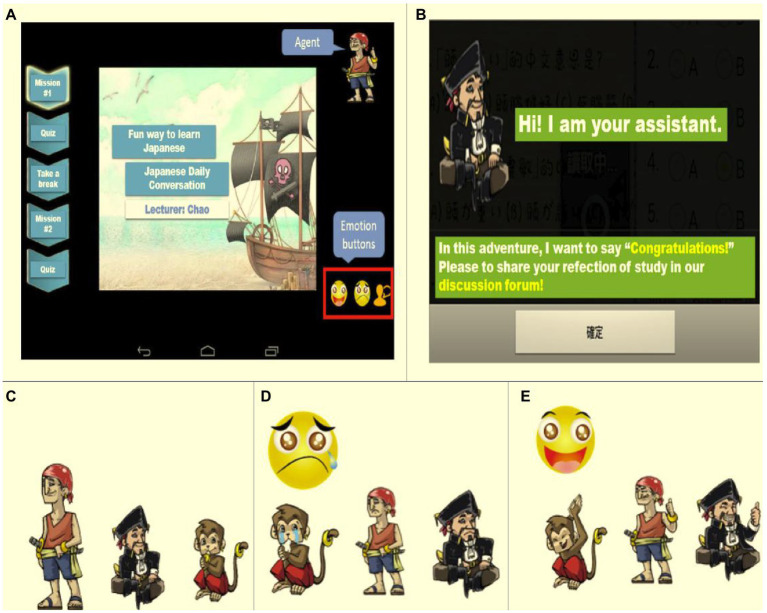
**(A)** Positive/negative emoticons on the course interface and emotion-related words for sentence formation. **(B)** Feedback at the end of the course. **(C)** Agent (from left to right): Sailor, shipmaster, monkey. **(D)** The agent’s response when a negative emoticon is selected. **(E)** The agent’s response when a positive emoticon is selected.

Upon completing tasks 1 and 2, learners have the option to express their thoughts and emotions, as shown in Figure 2, by selecting an emoticon (positive/negative) on the course interface and composing a sentence expressing the same. Based on the emotional cues obtained, the system provides a statically relevant emotional response through an agent. At the end of the course, the students’ emotional states during the learning activities and their levels of attention are evaluated, quiz scores calculated, and suggestions provided in the form of texts. Figure 2B illustrates the feedback provided at the end of the course, and Figures 2C–E shows the system agent design and feedback.

Students can choose to enter either the “Discussion Forum” or “Add New Issue” in ADF. To add a new issue, students enter the title and content related to the issue and select words that describe their emotions. Subsequently, by clicking on “reply,” learners can interact and engage in discussions with their peers asynchronously. This function enables learners to expand their knowledge and deepen their learning experience.

### Emotional Feedback Design

The learners select either positive or negative emotions and form a sentence by selecting words that describe their emotions. The selected words are then integrated to form a sentence according to which the agent interacts and responds.

Our emotional feedback module determines each student’s learning status and, consequently, whether or not the overall performance meets the course criteria. Our system has a critical value, called *maxClick* (maximum number of clicks), which is defined as the time (min) spent on learning a course multiplied by six. It reflects whether or not learners have paid attention in class, as shown in Equation (1). The overall judgment criteria are presented in Table 1.


(1) $$\mathrm{maxClick}=\mathrm{Duration}\;\left(\min\right)*6$$

**Table 1:** Rules for determining learners’ state during learning.

**State**	**Condition**
Unfocused	Number of times learners clicked on a positive emoticon (two units) + Number of times learners clicked on a negative emoticon (two units)
	> *maxClick*
Nervous	Number of times learners clicked on a positive emoticon (two units) + Number of times learners clicked on a negative emoticon (two units) > *maxClick*
	**OR**
	Number of times learners clicked on a positive emoticon (two units) + Number of times learners clicked on a negative emoticon (two units) < 1
Emo_Pos	Number of times learners clicked on a positive emoticon (two units) ≥
	Number of times learners clicked on a negative emoticon (two units)
Emo_Neg	Number of times learners clicked on a negative emoticon (two units) ≥
	Number of times learners clicked on a positive emoticon (two units)
Emo_No_Click	Number of times learners clicked on a negative emoticon (two units) < 0
	**AND**
	number of times learners clicked on a positive emoticon (two units) < 0
**Rules for determining learners’ state during learning: Test results**	
Grade_Pass	Test results for both tasks were ≥ 60
Grade_One_Pass	One of the test results was ≥60
Grade_Two_Fail	Test results for both tasks were ≤ 60
**Rules for determining positive/negative emotions**	
1. Set *kimo* as the variable for acquiring the emotion menu field address. Set *emotion* as the variable for acquiring the positive/negative emotion results. Set *emo_id* as the variable to access conversion of *kimo* values into strings. Set *sum_emo* as the variable to access conversion of *emotion* values into strings. 2. long kimo = parentView1.getItemIdAtPosition(position); long emotion = kimo%2; 3. emo_id = Long.toString(kimo); sum_emo = Long.toString(emotion) 4. Send *emo_id* and *sum_emo* as record	

**Figure 3:**
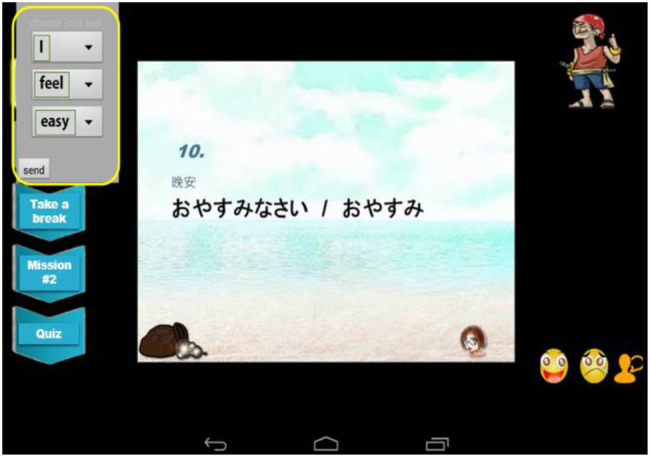
Screenshot of our ATS and learning environment.

Learners can choose from an assortment of words and phrases designed within the system to form a sentence that describes their emotions toward the course during the learning process. The sentence contains a subject (“I/This class”), verb (“is very/think”), and a word that reflects their feelings about the class, as shown in Figure 3.

The words were obtained from a national university in the authors’ country and included the following: “simple,” “difficult,” “cute,” “annoying,” “fun,” “frustrated,” “happy,” “sad,” “interesting,” “boring,” and words describing the course or the system. These words were categorized as positive and negative words and placed in a dropdown menu on the upper left corner of the system. The odd number and even number fields in the menu were calculated, and the results of the words selected by learners were computed. The sequence of the odd and even numbers corresponding to positive and negative emotions was 0, 1, 2, 3, etc. Once the stage was determined, *kimo* was set as the variable for acquiring the emotion menu field address; *emotion* was set as the variable for acquiring the results for positive/negative emotion words; *emo_id* was set as the variable to access the conversion of *kimo* values into strings; and *sum_emo* was set as the variable to access the conversion of *emotion* values into strings. Subsequently, two remaining values, 0 or 1, were obtained to determine whether a learner’s emotion had been positive or negative. Finally, *emo_id* and *sum_emo* were returned to the database at the server end and stored as the basis for recording students’ emotional events. The judgment rules are listed in Table 1. The system also records the sentences formed by the learners to describe their feelings about the course.

Various kinds of feedback for the emotions generated are provided at the end of the course according to (a) the rules established in the emotion recognition design and (b) the type of emotion (positive or negative). The system recorded the number of times learners clicked on a positive or negative emoticon during the course, their test results, and the sentences formed by them describing their feelings.

### Questionnaire Design

A cross-verification triangulation method was adopted for this study, which included a questionnaire, interviews, and observations to elucidate how learners felt about using AMLTS, including the ADF interface, the benefits of learning, and the measurement instruments employed. To evaluate whether students’ learning outcomes had increased on using the system, a learning outcome questionnaire was used as the research instrument. Two learning outcome questionnaires, a pretest and a posttest, were administered. The questionnaire comprised 30 multiple-choice questions on basic Japanese. The pretest and posttest questionnaires contained 30 multiple-choice items, for which the total score was 30. The ADF usability scale was evaluated and revised by three experts.

## Results

### Prototype Evaluation and Formal Evaluation Experiments

The participants in the first-phase prototype comprised 67 students who had never attempted to learn Japanese. The experiment was conducted in an actual classroom for approximately 50 to 100 min. During the prototype evaluation, the course was taught in two sessions in four sections: preschool guided reading, rules of writing the *goujon*, everyday language, and teaching Japanese numerals. The learning environment used in this experiment is shown in Figure 2.

A total of 63 students were invited to participate in the formal evaluation, including 38 learners who had participated in the prototype evaluation and 25 who had never used AMLTS. The course was designed in the following sections: preschool guided reading, rules of writing the *goujon*, everyday language, teaching Japanese numerals, and simple expressions in Japanese. The participants were asked to complete a pretest learning outcome questionnaire before they attempted to operate the system. At the end of the system operation, the participants were invited to an open interview. The posttest was conducted 1 month after the system operation.

### Learning Outcome Evaluation

The research results showed that on eliminating invalid samples from both groups of participants (those who had/had not participated in the prototype evaluation), the pretest results of Group 1 and Group 2 were 11.71 ± 6.32 and 13.80 ± 6.29, respectively. The results of the independent *t*-test were not significant (*t* = −1.203(*df* = 51), value of *p* = 0.24), suggesting that the knowledge levels of both groups before the experiment did not differ significantly. The posttest results for Group 1 and Group 2 were 23.04 ± 5.93 and 21.36 ± 5.58, respectively. The *t-*test results were not significant (*t* = 0.005, *p* = 0.99), suggesting that the posttest results of both groups did not differ significantly.

A paired-sample *t*-test was conducted on the differences between the pretest and posttest results for each group. The pretest and posttest results for group 1 differed by 11.321 ± 7.74. The average pretest and posttest results differed significantly (*t* = 7.74, value of *p* = 0.000), indicating that the pretest and posttest results of the participants who had participated in the prototype evaluation differed significantly. The pretest and posttest results for Group 2 differed by 7.56 ± 6.22. The average pretest and posttest results differed significantly (*t* = 6.08, value of *p* = 0.000), indicating that the pretest and posttest results of the participants who had not participated in the prototype evaluation differed significantly. Overall, the results showed that irrespective of their participation in the prototype evaluation, both groups of participants significantly improved in their performance through the use of AMLTS. The average difference between the pretest and posttest results of both groups was as follows: Levene = 2.463 (value of *p* = 0.122) and *t*-test = 0.008 (value of *p* = 0.996). Therefore, the pretest and posttest results of both groups of participants differed significantly, where both groups improved their learning outcomes.

### Emotion Feedback Analysis

The AMLTS recorded the number of times the study participants clicked on an emoticon, the sentences formed to express their emotions, their learning progress, their learning status, the test results, the activities (e.g., painting or listening to music and the song/music selected) learners chose during their break, new issues added in the discussion forum, and the learners’ responses to these issues. Research results indicated that some of the participants repeatedly took the course after watching the videos (dynamic) and reviewing the course content (static), and they practiced the quizzes until they passed them. In contrast, a few participants did not finish watching the videos and reviewing the course content, but they proceeded to learn the course and attempted to answer questions directly. The system provides three sessions of a course in the formal evaluation phase. All participants were able to complete the course. It was found that the course content was relatively simple for the participants, enabling learners to proceed faster, straight into the quiz.

The emotion event analysis showed that the learners clicked on emotions 527 times (positive: 274; negative: 40; no: 213) during the learning process. The learners did not click on any emoticons, suggesting that the material and the system helped the participants stay positive, while they used the system.

The positive words reflecting users’ feelings included “simple,” “fun,” “interesting,” “happy,” and “cute,” of which “simple” was the most commonly used word. Negative words included “difficult,” “frustrated,” and “boring,” of which “boring” was the most frequently used word in a total of 139 records. Positive sentences reflecting learners’ feelings about the course included, “I think [this course] is easy” and “This class is very easy.” An example of a negative sentence was “I think [this course] is boring.” Regarding the stress relief options provided by the application, participants favored listening to music over painting when taking a break and also preferred painting at the end of class for stress relief.

### Qualitative Interview Result Analysis

In the prototype evaluation phase, an open interview was conducted involving an instructor, a student operation instructor, and five participants. First, the participants suggested conferring rewards on passing the course or adding new games. Second, the participants suggested giving hints or providing simpler questions to help learners pass the course. The final suggestion was that the download speed and sound of the video could improve. In the interview in the formal evaluation phase, they suggested having the agents interact dynamically with users; for example, when a positive emoticon is selected, the agent could respond with, “That’s great!” or interact in different ways (*P1 and P2*). The ADF facilitated learning and allowed students to obtain solutions from their peers or to directly ask for help to quickly understand different areas (*all participants*).

### Evaluation of System Usability

Usability testing was adopted to test the system’s usability among 55 students in the ATS group after they had used the system. The negatively worded items in the questionnaire were modified to contain positive wordings. The results showed that the α value of the usability scale in the ATS group was 0.787, which exceeded the optimal reliability standard of 0.7 (Bangor, 2009), indicating that the results were satisfactorily reliable. Research results showed that most participants found the final system to be well-integrated and easy to use. Meanwhile, the users felt confident when using the system with the designed user interface.

All scores for the negative questions were modified to the scores for the positive questions. The mean score for learner satisfaction with the prototype system was 61.62, with a median of 66, a minimum of 50, a maximum of 90, and a standard deviation of 9.24. The mean score for learner satisfaction with the final system was 70.97, with a median of 68, a minimum of 44, a maximum of 100, and a standard deviation of 13.81. The results indicated that the scores for user satisfaction with the final system exceeded the median, implying that the users were moderately satisfied. In addition, the SUS score of the final system outperformed that of the prototype system.

## Conclusion and Discussion

Our AMLTS affective interaction design significantly improves learner engagement, collaborative discussion, and language learning performance, which is consistent with previous studies (Li et al., 2018; Mosa et al., 2018; Bakeer and Abu-Naser, 2019; Tafazoli et al., 2019; Demir, 2020). The results indicated that both the pretest and posttest results were significantly improved in the AMLTS. The results confirmed that affective factors and emotion feedback mechanisms were beneficial to engagement and performance (Jagger, 2013; Ziai et al., 2018; Demir, 2020).

The evaluation of system usability results showed that the learners were satisfied with and highly accepting of the AMLTS. They expressed satisfaction that the function and content were well-integrated, enhancing ease of use. The well-designed system helped students deepen their understanding of the learning content, easily understand the content, and engage in peer interaction, which increased their motivation (Mosa et al., 2018). When engaged in the discussion forum, the participants were inclined to raise questions about the course content, and most of them preferred adding a new issue and discussing it with their peers. However, current data show that the participants, overall, preferred responding to issues, and only a few participants added new ones. A system designer can modify courses in existing AMLTS into a system tailored to the needs of instructors (i.e., for use by learners), such as providing greater multimedia feedback during the course. Based on the findings of the research results, future studies could apply AMLTS in education that investigates the relationship between attitude and academic performance over a longer period of observation time and on a wider sample population. Finally, the limitations of the study include the small sample size, the representative of the sample that was only applied to university students, the type of non-probabilistic, and the absence of a control group. Such factors may be considered in future studies.

## Data Availability Statement

The original contributions presented in the study are included in the article, further inquiries can be directed to the corresponding authors.

## Author Contributions

All authors listed have made a substantial, direct and intellectual contribution to the work, and approved it for publication.

## Funding

This project was funded by the Ministry of Science and Technology (MOST), of the Republic of China under Contract numbers MOST 108-2511-H-142-007-MY2, MOST 110-2511-H-142-008-MY2, and 110-2511-H-006-012-MY3.

## Conflict of Interest

The authors declare that the research was conducted in the absence of any commercial or financial relationships that could be construed as a potential conflict of interest.

## Publisher’s Note

All claims expressed in this article are solely those of the authors and do not necessarily represent those of their affiliated organizations, or those of the publisher, the editors and the reviewers. Any product that may be evaluated in this article, or claim that may be made by its manufacturer, is not guaranteed or endorsed by the publisher.
